# A Geographically Sensitive Neighborhood Exposome–Wide Association Study for Breast Cancer Survival

**DOI:** 10.1001/jamanetworkopen.2025.58256

**Published:** 2026-02-18

**Authors:** Joseph Boyle, Hua Zhao, Kathryn Hughes Barry, Jie Shen, Carrie A. Miller, Cheryl L. Knott, Bernard F. Fuemmeler

**Affiliations:** 1 Department of Family Medicine and Population Health, Virginia Commonwealth University, Richmond; 2 Massey Comprehensive Cancer Center, Richmond, Virginia; 3 Department of Public Health Sciences, School of Medicine, University of Virginia, Charlottesville; 4 Program in Oncology, University of Maryland Greenebaum Comprehensive Cancer Center, Baltimore; 5 Department of Epidemiology and Public Health, School of Medicine, University of Maryland, Baltimore; 6 Department of Behavioral and Community Health, University of Maryland-College Park, College Park

## Abstract

**Importance:**

Growing literature recognizes the importance of contextual influences on outcomes across the cancer continuum, beyond those of individual biological and lifestyle factors. These contextual influences represent a neighborhood exposome consisting of socioeconomic, demographic, and built environmental factors.

**Objective:**

To identify which neighborhood-level factors are most relevant for cancer outcomes and determine the geographic scale of their operation.

**Design, Setting, and Participants:**

This cohort study of women with a primary diagnosis of breast cancer developed a geographically sensitive neighborhood exposome–wide association study (gs*-*NWAS) framework to determine specific neighborhood-level factors that may be associated with outcomes beyond individual-level factors. The study was performed at the University of Virginia Comprehensive Cancer Center from January 2014 to December 2024.

**Exposures:**

An analysis framework that assigned many neighborhood exposome components at 3 geographic scales: census tract and 1- and 5-km area-weighted buffer. Individual-level confounders (race and ethnicity, alcohol use, tobacco use, and body mass index) were also considered.

**Main Outcomes and Measures:**

Survival among women diagnosed with breast cancer. The gs-NWAS chose each component’s most relevant scale of operation, determined factors adjusted for multiple comparisons associated with survival in adjusted Cox proportional hazards regression models, and used machine learning methods (elastic net regression) to validate and condense these components. Hazard ratios (HRs), 95% CIs, and *P* values were reported.

**Results:**

Participants included 2727 women diagnosed with breast cancer (median age, 66 [IQR, 57-75] years) followed up for a median of 1697 (IQR, 945-2536) days (330 Black [12%], 2227 White [82%], and 170 other or unknown race [6%]); known cancer stage at diagnosis included I in 1668 (61%), II in 741 (27%), and III in 301 (11%). Four neighborhood exposome factors were associated with shorter breast cancer survival: high housing cost among low-income households (HR, 1.02; 95% CI, 1.00-1.03; *P* = .02 [tract]), people who recently moved to their current residence (HR, 1.06; 95% CI, 1.02-1.10; *P* = .001 [5-km buffer]), people renting in crowded households (HR, 1.03; 95% CI, 1.02-1.05; *P* < .001 [tract]), and the number of public preschoolers (HR, 1.06; 95% CI, 1.02-1.10; *P* = .001 [1-km buffer]).

**Conclusions and Relevance:**

In this cohort study of women diagnosed with breast cancer, a gs-NWAS was developed and applied to determine key components of the neighborhood exposome and their geographic scales of operation, identifying neighborhood-level factors in housing, economic, and demographic domains associated with breast cancer survival. Future studies should validate these findings in different populations and tailor this framework to their respective populations.

## Key Points

**Question:**

Can a novel analytic framework uncover more information regarding the neighborhood exposome’s association with breast cancer survival?

**Findings:**

This cohort study of 2727 women diagnosed with breast cancer in Virginia developed a geographically sensitive neighborhood exposome–wide association study analytic framework to identify which specific neighborhood exposome indicators are relevant for cancer outcomes and determine the geographic scale of their operation. Four components, including housing cost and density, recent relocation, and the number of children enrolled in public preschools, were associated with shorter survival.

**Meaning:**

This framework provides precise insights on how the neighborhood exposome was associated with health outcomes, in particular financial and housing-related stress for breast cancer survival.

## Teaser

This cohort study uses a geographically sensitive neighborhood exposome–wide association study analytic framework to identify which of the myriad components of the neighborhood exposome are associated with survival in patients with breast cancer and the relevant geographic scale of each component.

## Introduction

Breast cancer remains a significant public health concern in the US and worldwide. In 2025, it drove the largest number of new cancer diagnoses and second-largest number of cancer deaths among women.^1^ Incidence rates for breast cancer have steadily increased throughout this century, some of which can be attributed to local-stage and hormone receptor–positive diagnoses.^2^ Despite this upward trend, there have been notable improvements in breast cancer mortality, although there remains unevenness, with Black women experiencing lower survival rates compared with White women,^1,3^ and similarly with individuals living in poverty having worse breast cancer outcomes.^4,5^ The increasing population of breast cancer survivors in the US and disparities in mortality underscore a call for greater attention to risk factors associated with survival outcomes.

Growing literature has connected neighborhood-level socioeconomic factors and breast cancer outcomes. For example, the Area Deprivation Index, a multidimensional composite of neighborhood-level variables in the education, income, employment, and housing quality domains, is associated with many adverse outcomes, including later stage of diagnosis, lower treatment adherence, and shorter survival.^6,7,8,9,10,11^ Other constructed indices of neighborhood disadvantage and disinvestment have been linked to shorter breast cancer survival and highlight the intersecting influences of individual- and neighborhood-level socioeconomic status.^5,12,13,14,15^ Notably, Black individuals are more likely to experience lower socioeconomic status and live in disadvantaged neighborhoods than White individuals.^16,17,18^ Based on these findings, while individual biological (eg, genetic ancestry-related) factors may relate to tumor aggressiveness, other modifiable environmental pathways, including neighborhood socioeconomic status, racial stress-related discrimination, and social support, may be more relevant and actionable drivers of breast cancer survival–related differences.^14,19,20^

There are numerous components of the neighborhood environment, collectively comprising a “neighborhood exposome”^21,22^ of its own that necessitates consideration of all its components to better understand how they influence health outcomes. One effort to do so resulted in a neighborhood exposome–wide association study (NWAS) by Lynch et al.^23^ The NWAS used a multiphase framework based on informatics principles underlying the genome-wide association study that applied progressively more stringent criteria to filter out and group variables related to aggressive prostate cancer incidence. This framework evaluated thousands of neighborhood environment components and provided an impressive increase in specificity of findings from general single-number neighborhood disadvantage indices to specific census-tract level components and groups of the neighborhood environment. The authors also acknowledged the need for future NWAS-related studies to adjust for individual socioeconomic status and other factors and evaluate the effects of boundary selection choices for geospatial variables. Regarding the former, it is important when possible to account for individual-level variables that simultaneously impact health outcomes^24^ (eg, for breast cancer, lifestyle behaviors, body mass index [BMI], etc). Regarding the latter, including components of the neighborhood environment across multiple spatial boundaries (eg, census tract defined by the Census Bureau, zip code defined by the US Postal Service, residential buffers defined by an individual’s home and given buffer radius) is crucial for several reasons: individuals themselves may live proximate to an administrative boundary; components may be relevant only close to the residence or over a larger area; and measures taken over different distances may better represent their influence on individuals’ lives. These various levels of geospatial boundary selection may vary with regard to interpretability or administrative oversight and clinical assumptions encoded in their use. Addressing these 2 factors is essential for gaining a deeper understanding of the complex, intersecting relations among individual- and neighborhood-level variables and cancer outcomes.

Therefore, this cohort study aimed to develop a geographically sensitive NWAS (gs-NWAS) to identify which of the myriad components of the neighborhood exposome are associated with breast cancer survival outcomes and the relevant geographic scale of each component. We expected that our gs-NWAS framework would identify a subset of neighborhood exposome components that were associated with breast cancer survival above and beyond those of individual factors and that the components would operate at unique geographic scales. Such results would offer insight into underlying mechanisms and provide clues for future policy interventions to promote improved and equitable breast cancer survivorship.

## Methods

### Data Source

We obtained data from a cohort of women diagnosed with breast cancer at the University of Virginia Comprehensive Cancer Center, Charlottesville, from January 2014 to December 2024. The initial cohort included 3041 patients. After excluding women with no residential information, no specific date of diagnosis recorded, or fewer than 60 days from diagnosis to death, those who lived outside Virginia, or those whose address did not link to neighborhood variables, the remaining cohort included 2727 patients. We obtained information on survival outcomes (time until death, defined by the number of days between diagnosis and death or right censoring at September 23, 2024, whichever came first), clinical characteristics (cancer stage, triple-negative status, BMI), demographic characteristics (age, self-reported race [Black, White, or Other, including Asian, Native Hawaiian and Other Pacific Islander, and multiracial] and ethnicity [Hispanic or non-Hispanic]), and lifestyle behaviors (alcohol use, tobacco use). Due to the socioeconomic focus of our study, which has implications via the social construct of race and ethnicity, we recorded and included these factors in the analysis. We also obtained geocoded residential location at the time of diagnosis (latitude/longitude) using ArcGIS, version 10.8 (Environmental Systems Research Institute Inc)^25^ and census tract to link to the neighborhood exposome variables described below. The Institutional Research Board at the University of Virginia approved this study. Informed consent was waived for this use of retrospective data. We followed the Strengthening the Reporting of Observational Studies in Epidemiology (STROBE) reporting guideline.

### Neighborhood Exposome Assignment

Participants’ census tract information was used to link with numerous data sources to comprise a neighborhood exposome. We performed linkages with 13 datasets, resulting in 5553 variables comprising the neighborhood exposome (from domains of the American Community Survey [ACS], Centers for Disease Control and Prevention PLACES, modeled concentrations of air pollutants, incarceration rate, historical redlining, land developed, alcohol binge drinking, no frequent physical activity, obesity, days with particulate matter less than 2.5 µm in diameter and ozone above regulatory thresholds, population living in close proximity to highways, and racial residential segregation index) across multiple geographic scales. For each variable, we assigned values at 3 geographic scales: census tract, 1-km area-weighted buffer, and 5-km area-weighted buffer. More information on these datasets and scale assignments can be found in the eMethods and eTable 1 in Supplement 1.

### Statistical Analysis

We used the following steps of the gs-NWAS to determine which components of the neighborhood exposome were associated with breast cancer survival, and their specific geographic scales of operation. As an initial filtering step, we removed variables having zero values for greater than 70% of the sample to prevent the influence of highly sparse, unstable, and/or irrelevant variables in the analyses. This excluded all geographic levels of the days above the regulatory threshold for particulate matter less than 2.5 µm in diameter and ozone, historical redlining, ethylene oxide–modeled concentration variables, and 1151 ACS variables. First, for each variable, we fit a separate mixed-effects Cox proportional hazards (PH) regression model at each geographic scale, with breast cancer survival in days as the outcome; race, ethnicity, tobacco use, alcohol use, and BMI as covariates; and using a random effect for census tract, given clustering of the participants within census tracts. We adjusted for these covariates based on constructing a directed acyclic graph (eFigure in Supplement 1) to estimate the direct effect of the neighborhood exposome on breast cancer survival. We determined the most relevant scale of operation for each variable by recording the scale with the smallest *P* value. Second, among the set of variables at their most relevant scale, we refit the mixed-effects Cox PH regression models and obtained false discovery rate–corrected significant (*q* < .10) variables from these. Third, we fit elastic net Cox PH regression models to filter the most significant and predictive neighborhood exposome components using a random 70%-30% training-testing split. We determined the mixing parameter α by testing a fine grid of values in (0, 1) with the training data, choosing the model with the highest C index. Given this α, we fit an elastic net Cox PH regression model to the testing data to choose coefficients. Fourth, with the set of variables chosen, we fit a full mixed-effects Cox PH regression model with the covariates and random effects above. We reported hazard ratios (HRs), *P* values, and 95% CIs and checked the PH assumption via score tests of time-dependent coefficients for each variable. Additionally, we conducted 3 sensitivity analyses (1) additionally adjusting for participant-level insurance, marital, and employment status; (2) additionally adjusting for age at diagnosis; and (3) additionally adjusting for stage at diagnosis. The third analysis identified violation of PH in stage and worse model fit and thus results of this model are not reported. We also created a neighborhood risk index by combining significant identified neighborhood components, weighted by their regression coefficients, testing its association with survival in the full mixed-effects adjusted Cox PH regression model. Hypothesis tests were 2 sided, and *P* < .05 indicated statistical significance. We conducted all analyses in R, version 4.5.0 (R Project for Statistical Computing).

## Results

Participants included 2727 women diagnosed with breast cancer (median age, 66 [IQR, 57-75] years) followed up for a median of 1697 (IQR, 945-2536) days. Of these, 330 women (12%) were Black, 2227 (82%) were White, and 170 (6%) of other or unknown race. Cancer stage at diagnosis included stage I in 1668 patients, (61%), stage II in 741 (27%), stage III in 301 (11%), and unknown in 17 (1%); 100 (4%) were Hispanic. Characteristics of women diagnosed with breast cancer are provided in Table 1. Four hundred forty-three women (16%) died by the end of follow-up, with a median of 989 (IQR, 454-1618) days to death. Among those who had not died, a median of 1848 (IQR, 1066-2625) days to censoring occurred. One hundred ninety-two women (7%) had triple-negative status. Ever alcohol use was reported by 1530 women (56%) and ever tobacco use by 1113 (41%).

**Table 1.** Summary of Clinical, Demographic, and Lifestyle Behavior Characteristics of Individuals Diagnosed With Breast Cancer

Characteristic	No. (%) of patients (N = 2727)
Deaths	443 (16)
Time from diagnosis to death for participants who died, median (IQR), d	989 (454-1618)
Time from diagnosis to death for censored participants, median (IQR), d	1848 (1066-2625)
Stage at diagnosis	
I	1668 (61)
II	741 (27)
III	301 (11)
Unknown	17 (1)
Triple-negative status	
No	2211 (81)
Yes	192 (7)
Unknown	324 (12)
BMI, median (IQR)	28.1 (23.7-32.9)
Age, median (IQR), y	66.0 (57.0-75.0)
Race	
Black	330 (12)
White	2227 (82)
Other^a^	163 (6)
Unknown	7 (0.3)
Ethnicity	
Hispanic	100 (4)
Non-Hispanic	2617 (96)
Unknown	10 (0.4)
Alcohol use	
Ever	1530 (56)
Never	1153 (42)
Unknown	44 (2)
Tobacco use	
Ever	1113 (41)
Never	1587 (58)
Unknown	27 (1)

Abbreviation: BMI, body mass index (calculated as weight in kilograms divided by height in meters squared).

^a^ Includes Asian, Native Hawaiian and Other Pacific Islander, and multiracial.

The gs*-*NWAS initially considered 5553 neighborhood exposome components (1851 components at 3 scales). The geographic scale estimation step identified 461 variables as operating optimally at the tract level, 405 at the 1-km buffer level, and 734 at the 5-km buffer level. Of these, 193 had significant uncorrected *P* values, all but one from the ACS or modeled air pollutant concentration domains (Figure, A). Ten variables had false discovery rate–corrected *q* values of less than .10 and entered the filtering step, all from ACS (Table 2 and Figure, B). The elastic net regression step using optimal identified hyperparameters selected 7 variables among this set (Table 2) to be included in the final, multivariable-adjusted Cox PH regression model.

**Figure.**
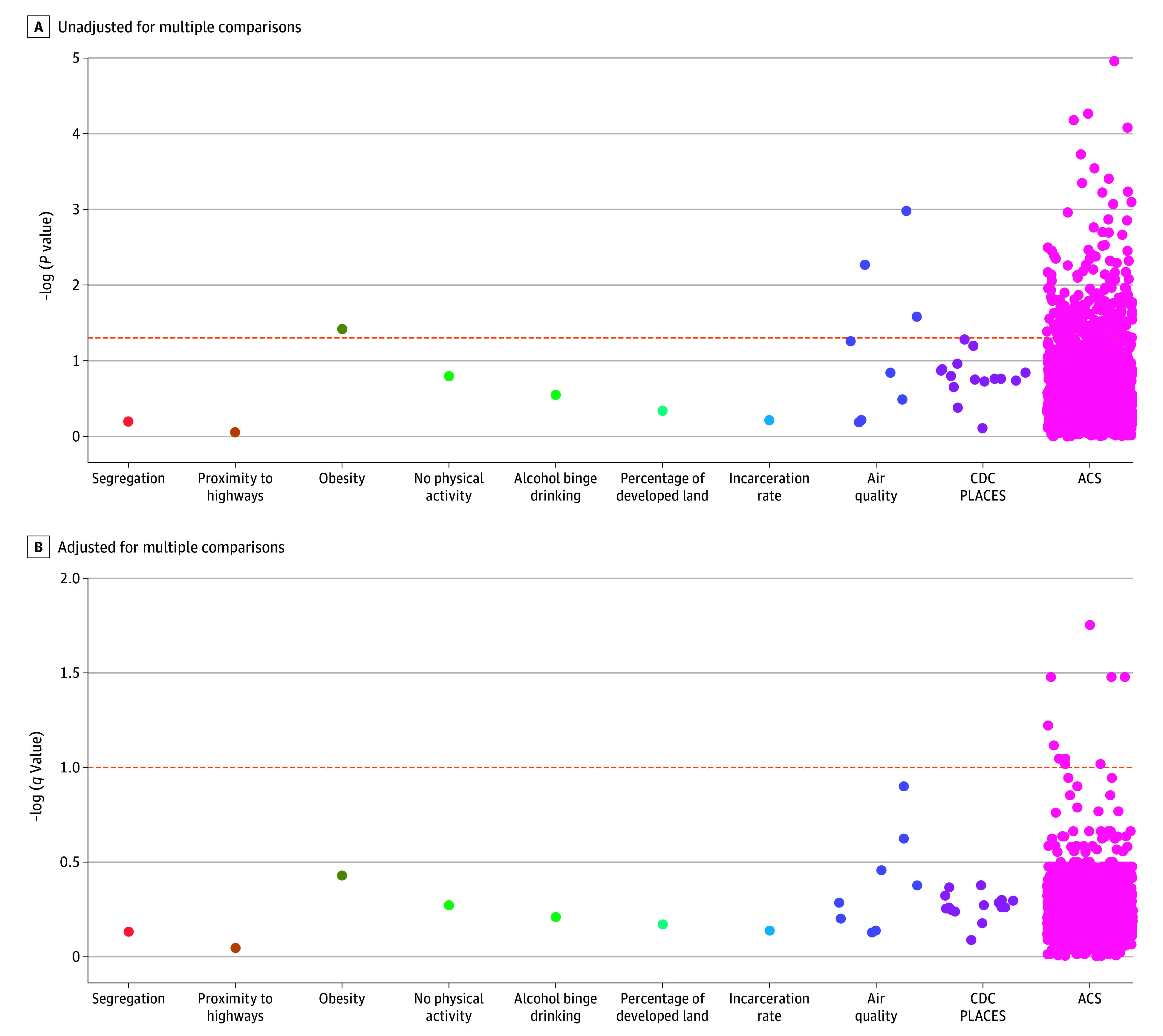
Manhattan Plot of Geographically Sensitive Neighborhood Exposome–Wide Association Study Components and Breast Cancer Survival The y-axis displays the negative logarithm of *P* and *q* values, so higher points along this axis denote smaller *P* and *q* values. A, The dashed orange line corresponds to *P* = .05; points above this line denote associations with breast cancer survival unadjusted for multiple comparisons. B, The dashed orange line corresponds to a multiple comparisons-adjusted *q* value of .10; points above this line denote associations with breast cancer survival adjusted for multiple comparisons. ACS indicates American Community Survey; CDC, Centers for Disease Control and Prevention.

**Table 2.** Neighborhood Exposome Components With *q* Values Less Than .10 in Initial Steps of Geographically Sensitive Neighborhood Exposome–Wide Association Study

Exposome factor	Geographic scale	*P* value	*q* Value^a^	Selected by elastic net step^b^
Percentage of renter-occupied households paying >30% of household income and earning $35 000 to $49 999 per year	Tract	<.001	.02	Yes
Percentage of occupied housing units among households paying >30% of household income and earning $35 000 to $49 999 per year	Tract	<.001	.03	Yes
Percentage of occupied housing units moving in 2021 or later	5-km Buffer	<.001	.03	Yes
Percentage of renter-occupied housing units moving in 2021 or later	5-km Buffer	<.001	.03	No
Percentage of people reporting Brazilian ancestry among multiple ancestries	5-km Buffer	<.001	.06	Yes
Percentage of renter-occupied housing units with householder aged 25 to 34 y	1-km Buffer	<.001	.08	No
Percentage of total population reporting Brazilian ancestry	5-km Buffer	<.001	.09	Yes
Percentage of population 3 y and older enrolled in public preschool	1-km Buffer	<.001	.09	Yes
Percentage of renter-occupied housing units with 6-person household	Tract	<.001	.10	Yes
Percentage of total population in renter-occupied housing units moving in 2021 or later	5-km Buffer	<.001	.10	No

^a^ Represents a false discovery rate-corrected Benjamini-Hochberg *P* value.

^b^ Identified optimal parameters of α = 1/30 and λ = 0.3594.

Four neighborhood exposome factors were associated with significantly shorter breast cancer survival in the final model (Table 3). Specifically, these were the percentage of residents paying more than 30% of household income on housing costs among renter-occupied households earning $35 000 to $49 999 per year within tract (high housing cost–low income; HR, 1.02 [95% CI, 1.00-1.03]; *P* = .02); the percentage moving into the current household in the most recent year among occupied housing units within 5-km buffer (recent movers; HR, 1.06 [95% CI, 1.02-1.10]; *P* = .001); the percentage of households containing 6 or more people among renter-occupied housing units within tract (crowded renters; HR, 1.03 [95% CI, 1.02-1.05]; *P* < .001); and the percentage enrolled in preschool among the population 3 years or older enrolled in public school within 1-km buffer (public preschoolers; HR, 1.06 [95% CI, 1.02-1.10]; *P* = .001). The PH assumption was satisfied as the time-dependent coefficients for every variable were nonsignificant.

**Table 3.** Summary of Neighborhood Exposome Components and Covariates in Final Geographically Sensitive Neighborhood Exposome–Wide Association Study Model of Breast Cancer Survival^a^

Exposome factor	Geographic scale	HR (95% CI)	*z* Value	*P* value
Percentage of renter-occupied households paying >30 percent of household income and earning $35 000 to $49 999 per year	Tract	1.02 (1.00-1.03)	2.41	.02
Percentage of occupied housing units paying >30% of household income and earning $35 000 to $49 999 per year	Tract	1.02 (0.99-1.06)	1.16	.24
Percentage of occupied housing units moving in 2021 or later	5-km Buffer	1.06 (1.02-1.10)	3.20	.001
Percentage of people reporting Brazilian ancestry among multiple ancestries	5-km Buffer	1.32 (0.79-2.21)	1.07	.29
Percentage of total population reporting Brazilian ancestry	5-km Buffer	0.57 (0.10-3.23)	−0.64	.53
Percentage of renter-occupied housing units with 6-person household	Tract	1.03 (1.02-1.05)	4.11	<.001
Percentage of population 3 y and older enrolled in public preschool	1-km Buffer	1.06 (1.02-1.10)	3.29	.001

Abbreviation: HR, hazard ratio.

^a^ Indicates results from mixed-effects Cox proportional hazards regression model with census tract as random effect and adjusting for race, ethnicity, body mass index, and alcohol and tobacco use.

Four of 6 pairwise correlations between identified neighborhood exposome components were nonsignificant. The exceptions were high housing cost–low income with recent movers (ρ = 0.18; *P* < .001) and with crowded renters (ρ = −0.06; *P* < .001), indicating that collinearity between significant neighborhood exposome components did not pose a major issue.

The combined neighborhood risk index was associated with significantly shorter survival (HR, 1.18; 95% CI, 1.11-1.25; *P* < .001). In both sensitivity analyses, all identified components remained associated with significantly shorter survival; associations did not attenuate in magnitude.

## Discussion

In this cohort study, we developed a novel analytic framework to better understand the geographically varying association of the neighborhood exposome with breast cancer survival, the gs-NWAS. We illustrated how, by using this framework, it is possible to determine which of a litany of indicators in the neighborhood exposome is associated with a health outcome of interest, as well as the geographic scale on which it influences the outcome. Our methodology found that 4 neighborhood exposome components were associated with shorter survival in patients with breast cancer: high housing cost–low income within census tract, recent movers within the 5-km buffer, crowded renters within census tract, and public preschoolers within the 1-km buffer. These components, which can represent various embodiments of financial stress, warrant follow-up in future studies of breast cancer survival.

Regarding specific findings of our analysis, several results align with existing literature, while others identify novel area-level factors operating at unique geographic scales that play important roles in breast cancer survival. Two recent studies examining neighborhood-level components provide valuable points of comparison.^5,15^ One analysis of SEER registry data found that residing in areas of persistent poverty^4^ was associated with increased breast cancer–specific and all-cause mortality.^5^ Another study of a population-based cohort of breast cancer survivors found that neighborhood disinvestment was associated with shorter survival among individuals diagnosed at moderate stages (II or III).^15^

In assessing census tracts with the highest values across 4 identified neighborhood exposome components (eTable 2 in Supplement 1), almost all were not considered areas of persistent poverty according to the most recent report.^26^ Moreover, most were located in nonurban areas, whereas urban areas tend to have larger values according to this disinvestment measure.^15^ Therefore, several of our findings may be considered novel neighborhood-level risk factors for breast cancer survival, representing independent dimensions beyond those previously identified. For example, paying a disproportionate amount of household income for housing among low-income renting households parallels an inability to secure homeownership. Research has linked other neighborhood-level indicators such as mortgage rate denial and racial bias in mortgage lending with increased breast cancer-specific mortality.^27,28,29,30^ Regarding recent movers, a high degree of residential mobility has been identified in cancer survivors,^31^ driven by various factors, including proximity to family members, treatment facilities, and/or losing or changing occupations after diagnosis. Residential crowding, another component, contributes to chronic stress,^20,32,33^ a condition that may shorten survival through inflammation and systemic dysregulation. For example, both objective (geospatial) and subjective (individually perceived) measures of neighborhood disadvantage have been correlated with biological markers of aggressive breast cancer that are associated with shorter survival.^34^

As an analytic framework, the gs-NWAS provides a powerful approach in the era of big data, enabling a fuller account of the influence of contextual factors on health. Despite growing evidence supporting the role of neighborhood-level factors on outcomes across the breast cancer continuum,^5,6,7,8,9,10,11,12,13,14,15^ there remains a dearth of social epidemiologic research in relation to population health outcomes relative to dominant biomedical and lifestyle behavior theories of disease distribution.^35^ This gap presents a significant opportunity for continued attention to the role of external factors for health outcomes and inequities, and more specifically, the development and application of modeling frameworks that support this view.

### Strengths and Limitations

To this end, the gs*-*NWAS has several attractive features. First, it is agnostic along 2 dimensions—specific neighborhood exposome components and specific geographic scales of operation—allowing the data to inform both dimensions. This stands in contrast to precalculated summary measures such as the Area Deprivation Index and allows more granular understanding of the specific neighborhood exposome components driving health outcomes. It also improves on typical geographic exposure assignment: assigning a value based on the administrative boundary, such as census tract, containing the observation. While the gs-NWAS has census tract as a possibility, it also considers area-weighted geographic buffers to uncover the most relevant geographic scale of operation for a variable. This attenuates the modifiable area unit problem^36^ by considering multiple continuous measures of geographic proximity. Second, through its derived neighborhood risk index, the method can support risk stratification or target neighborhoods for screening and resource allocation.^23^ Third, it retains elements of similarity to genome-wide and epigenome-wide association studies via including a filtering step to remove low-prevalence variables (filtering out probes with low detection frequency or single-nucleotide variants), quasi-replication through the elastic net regression test data step (validation of findings in different populations), and derived neighborhood risk index creation (polygenic risk score). Future work will extend the replication step to new populations to validate the role of neighborhood exposome components on breast cancer survival.

This study also has some limitations. First, although we included important participant-level data, we did not have all possible such variables and would have benefitted from treatment information. Relatedly, information for covariates such as BMI and alcohol use were obtained after diagnosis and may have been influenced by the disease. Second, the sample consisted of predominantly White individuals treated at a single academic medical center, limiting generalizability. Future research should increase geographic and racial and ethnic diversity, which could support validation exercises similar to those conducted in multiple racial groupings from NWAS findings.^37^ Third, our neighborhood exposome assignment used residential address at diagnosis, which may not reflect the contextual environment of individuals who move after their diagnosis. Fourth, certain variable domains were at county and not census-tract level owing to sample size limitations of the underlying datasets and to illustrate the ability of the gs-NWAS to handle data reported at multiple geographic scales. Future applications will use data linkages at the smallest geographic scale as appropriate that result in stable estimates. Fifth, our approach may result in finding important neighborhood exposome components that operate across residential buffers that may cross administrative boundaries, such as counties. In this case, intercounty collaboration may be required to correctly allocate resources and enact public health interventions,^38^ or if this is infeasible or impractical, weighing the difference in explanatory power that could result from considering the simpler geographic scale, such as census tract level operation. However, prevalence of multicounty buffers was relatively scant in our sample. Last, the methodology’s model selection approach may not have identified the precise causal pathways underlying contextual factors for breast cancer survival (and may exhibit attenuated effect estimates due to adjusting for potentially mediating factors) and will benefit from validation in different and larger populations and incorporation of additional contextual variables in different domains (eg, physical environmental characteristics, including sidewalk walkability^39^ and neighborhood disinvestment^40^) to more completely characterize the neighborhood exposome.

## Conclusions

In this cohort study of women diagnosed with breast cancer, we introduced a new analytic framework for examining large-scale neighborhood-level data in relation to cancer outcomes. This framework can be applied to any health outcome where the neighborhood exposome is hypothesized to play a role. In this study, 4 neighborhood-level variables were associated with shorter breast cancer survival, above and beyond individual-level factors. Future research should aim to validate these findings across different populations and further refine the framework as necessary to better understand the role of contextual variables on outcomes across the cancer continuum.

## Notes

Supplement 1. **eMethods.** Data Source and Neighborhood Exposome Assignment **eTable 1.** Summary of Data Sources for Geographically Sensitive Neighborhood Exposome–Wide Association Study Components **eTable 2.** Census Tracts Having the Highest Values of Identified Neighborhood Exposome Components Associated With Shorter Breast Cancer Survival **eFigure.** Directed Acyclic Graph for the Association Between the Neighborhood Exposome and Breast Cancer Survival
eReferences

Supplement 2.
Data Sharing Statement
