# Supplementary material for: A Geographically Sensitive Neighborhood Exposome–Wide Association Study for Breast Cancer Survival
Source: JAMA Netw Open. 2026 Feb 18;9(2):e2558256. doi: 10.1001/jamanetworkopen.2025.58256 (PMC12917675; doi:10.1001/jamanetworkopen.2025.58256)
Supplement: Supplement 1. — eMethods. Data Source and Neighborhood Exposome Assignment eTable 1. Summary of Data Sources for Geographically Sensitive Neighborhood Exposome–Wide Association Study Components eTable 2. Census Tracts Having the Highest Values of Identified Neighborhood Exposome Components Associated With Shorter Breast Cancer Survival eFigure. Directed Acyclic Graph for the Association Between the Neighborhood Exposome and Breast Cancer Survival eReferences [file jamanetwopen-e2558256-s001.pdf]

Supplementary Online Content

Boyle J, Zhao H, Barry KH, et al. A geographically sensitive neighborhood exposome-wide association study for breast cancer survival. *JAMA Netw Open.* 2026;9(2):e2558256. doi:10.1001/jamanetworkopen.2025.58256

eMethods. Data Source and Neighborhood Exposome Assignment

eTable 1. Summary of Data Sources for Geographically Sensitive Neighborhood Exposome-Wide Association Study Components

eTable 2. Census Tracts Having the Highest Values of Identified Neighborhood Exposome Components Associated With Shorter Breast Cancer Survival

eFigure. Directed Acyclic Graph for the Association Between the Neighborhood Exposome and Breast Cancer Survival

eReferences

This supplementary material has been provided by the authors to give readers additional information about their work.

© 2026 Boyle J et al. *JAMA Network Open*.

eMethods. Data Source and Neighborhood Exposome Assignment

Data Source. Breast cancer was identified as having ICD-10-CM codes C50.x. We obtained on survival outcomes from the institutional cancer registry, which uses data from the Virginia Cancer Registry as well as active follow-up. Stage was determined via the AJCC 8th edition. Triple-negative status was defined as negative for estrogen receptor, progesterone receptor, and human epidermal growth factor receptor. Body mass index was defined as weight in kilograms divided by squared height in meters. For geocoding in ArcGIS, we required a match score of at least 95. We treated loss to follow-up as right-censoring. UVACCC is the major cancer care provider for a large geographic area and therefore the majority of patients diagnosed there are treated and followed up at this institution. To minimize the loss to follow-up, the institution's cancer registry applies both passive follow-up (using data from the Virginia Cancer Registry based on physician-validated state death certificates and National Death Index data, following Commission on Cancer/SEER rules) as well as active follow-up by mailing postcards to cancer survivors.

Neighborhood exposome assignment. We performed linkages with the following datasets: racial residential segregation[1]; population living in close proximity to highways; days per year over the $PM_{2.5}$ and Ozone regulatory thresholds; prevalence of obesity; percent of population with no frequent physical activity or reporting alcohol binge drinking; percent of developed land; intersection with historically redlined areas[2]; incarceration rate; modeled concentrations of several air pollutants; behavioral risk factor variables from the CDC PLACES[3]; and all variables from the American Community Survey (ACS)[4].

The first scale was census tract, meaning that we assigned the value of the variable for the census tract containing the participant's residential location. The second scale was 1-kilometer (km) area-weighted buffer. Specifically, we created a circular buffer with radius 1 km around the participant's location and intersected it with census tract boundaries, using an average of the variable values that was weighted by the area overlap of the buffer with multiple census tracts intersecting it. The third scale was a 5-km area-weighted buffer calculated in the same manner as above. Certain variables were reported at the county level. In this case, we performed the process above, but with values assigned at county level. This process follows from spatial scale estimation[5,6], which acknowledges the need to evaluate the geographic scale of an exposure's operation.

eTable 1. Summary of Data Sources for Geographically Sensitive Neighborhood Exposome-Wide Association Study Components

**Variable Group**	**Geographic level**	**Time period**	**Source**	**Notes**
American Community Survey	Census tract	2014-2018, 2019-2023	Social Explorer	• 5454 variables<br>• All percentage-scale variables<br>• Excluded: 1151 variables
CDC PLACES variables	County	2021-2022	CDC PLACES	• 45 variables<br>• Behavioral-type variables (age-adjusted) from BRFSS: Arthritis among adults; Diagnosed diabetes among adults; Depression among adults; Current asthma among adults; Mobility disability among adults; Current cigarette smoking among adults; Frequent mental distress among adults; Visited dentist or dental clinic in the past year among adults; Chronic

© 2026 Boyle J et al. *JAMA Network Open*.

				obstructive pulmonary disease among adults; Coronary heart disease among adults; Visits to doctor for routine checkup within the past year among adults; Mammography use among women aged 50-74 years; Fair or poor self-rated health status among adults; Current lack of health insurance among adults aged 18-64 years; Short sleep duration among adults
Modeled concentrations of air pollutants	Census tract	2011-2019	EPA / National Air Toxics Assessment	• 24 variables<br>• Air pollutants: 1,3-butadiene, acetaldehyde, benzene, carbon tetrachloride, diesel particulate matter, ethylene oxide, formaldehyde, and naphthalene<br>• Excluded: ethylene oxide (1km, 5km, tract)
Incarceration Rate	Census tract	2020	Prison Policy Initiative	• 3 variables
Historical Redlining	Census tract	Dependent on times of historical redlining (generally 1930s)	Mapping Inequality Project	• 3 variables<br>• Included redlining maps from: Harrisonburg, Lynchburg, Roanoke, Staunton, Danville, Bristol, Charleston (WV)<br>• Excluded: Historical Redlining (1km, 5km, tract)
% of land developed	Census tract	2011-2021	National Land Cover Database	• 3 variables
**Variable Group**	**Geographic level**	**Time period**	**Source**	**Notes**
Alcohol binge drinking	County	2018-2021	Behavioral Risk Factor Surveillance System	• 3 variables<br>• % Reporting from BRFSS
No frequent physical activity	County	2018-2021	Behavioral Risk Factor Surveillance System	• 3 variables<br>• % Reporting from BRFSS
Obesity	County	2018-2021	Behavioral Risk Factor Surveillance System	• 3 variables<br>• % Reporting from BRFSS
Days over $PM_{2.5}$ regulatory threshold	Census tract	2014-2020	EPA / Environmental Public Health Tracking Network	• 3 variables<br>• Excluded: $PM_{2.5}$ (1km, 5km, tract)
Days over ozone regulatory threshold	Census tract	2014-2020	EPA / Environmental Public Health Tracking Network	• 3 variables<br>• Excluded: ozone (1km, 5km, tract)

© 2026 Boyle J et al. *JAMA Network Open*.

% of population living in close proximity to highways	Census tract	2010-2020	EPA / Department of Transportation	• 3 variables
Racial residential segregation index	Census tract	2014-2018, 2019-2023	“ndi” package in R	• 3 variables<br>• Anthopolos measure: ranges from 0-1, used non-Hispanic Black residential segregation measure

Notes: Abbreviations used are CDC (Centers for Disease Control and Prevention), PLACES (Population Level Analysis and Community Estimates), BRFSS (Behavioral Risk Factor Surveillance System), $PM_{2.5}$ (particulate matter less than 2.5 microns in diameter), and EPA (Environmental Protection Agency). For a given domain, the phrase “X variables” means that X/3 unique variables were included, with each assigned at three geographic scales. The “Excluded” bullet point lists the variables that were ultimately not considered in our data due to >70% of the values being zero. References for specific data sources are given in the eMethods.

© 2026 Boyle J et al. *JAMA Network Open*.

eTable 2. Census Tracts Having the Highest Values of Identified Neighborhood Exposome Components Associated With Shorter Breast Cancer Survival

Neighborhood exposome component	Tract	Municipality	Value (%)
High housing cost/low income	51069050402	Frederick	86
	51137110107	Orange	51
Recent movers [a]	51730810900 [c]	Petersburg city	17
	51035080200	Carroll	16
	51700031902	Newport News city	16
	51660000203 [c]	Harrisonburg city	16
Crowded renters	51059482604	Fairfax	100
	51067020103	Franklin	74
Public preschoolers [b]	51133020200	Northumberland	23
	51031020600	Campbell	20

Notes: [a] Scale for identified neighborhood exposome component was 5km buffer but value presented is for census tract. [b] Scale for identified neighborhood exposome component was 1km buffer but value presented is for census tract. [c] Area of Persistent Poverty for MPDG 2025-2026.

eFigure. Directed Acyclic Graph for the Association Between the Neighborhood Exposome and Breast Cancer Survival

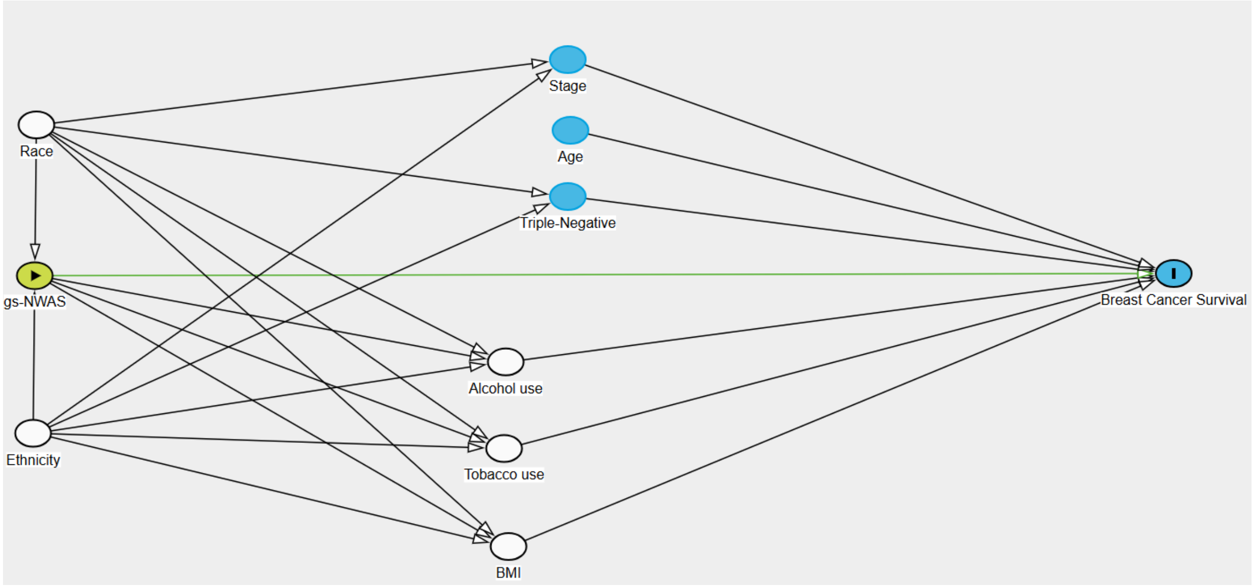

Notes: Green: exposure of interest; Blue with line: outcome; Blue without line: unadjusted; White: Adjusted. Abbreviations used: gs-NWAS: geographically sensitive neighborhood-wide association study; BMI: body mass index.

© 2026 Boyle J et al. *JAMA Network Open*.

eReferences

1. Anthopolos R, James SA, Gelfand AE, Miranda ML. A spatial measure of neighborhood level racial isolation applied to low birthweight, preterm birth, and birthweight in North Carolina. *Spat Spatio-Temporal Epidemiol*. 2011;2(4):235-246. doi:10.1016/j.sste.2011.06.002

2. Digital Scholarship Lab, University of Richmond. Mapping Inequality: Redlining in New Deal America. June 23, 2023. Accessed July 23, 2023. https://dsl.richmond.edu/panorama/redlining/

3. Centers for Disease Control. PLACES: Local Data for Better Health. CDC. 2025. Accessed July 8, 2025. https://www.cdc.gov/places/index.html

4. US Census Bureau. 2014-2018 American community survey 5-year estimates. Published online 2019.

5. Grant LP, Gennings C, Wickham EP, Chapman D, Sun S, Wheeler DC. Modeling pediatric body mass index and neighborhood environment at different spatial scales. *Int J Environ Res Public Health*. 2018;15(3). doi:10.3390/ijerph15030473

6. Grant LP, Gennings C, Wheeler DC. Selecting spatial scale of covariates in regression models of environmental exposures. *Cancer Inform*. 2015;14:CIN-S17302.

© 2026 Boyle J et al. *JAMA Network Open*.
