# Supplementary material for: A Geographically Sensitive Neighborhood Exposome–Wide Association Study for Breast Cancer Survival
Source: JAMA Netw Open. 2026 Feb 18;9(2):e2558256. doi: 10.1001/jamanetworkopen.2025.58256 (PMC12917675; doi:10.1001/jamanetworkopen.2025.58256)
Supplement: Supplement 2. — Data Sharing Statement [file jamanetwopen-e2558256-s002.pdf]

Data Sharing Statement

Boyle. A Geographically Sensitive Neighborhood Exposome–Wide Association Study for Breast Cancer Survival. *JAMA Netw Open*. Published February 18, 2026.
doi:10.1001/jamanetworkopen.2025.58256

Data
Data available: No

Additional Information
Explanation for why data not available: Data are not shared due to confidentiality reasons. Data inquiries should be directed toward the University of Virginia Office of Sponsored Programs. To gain access to the data, requesters will need to sign a data access agreement with the University of Virginia.
